# Mechanism of the Impact-Sensitivity Reduction of Energetic CL-20/TNT Cocrystals: A Nonequilibrium Molecular Dynamics Study

**DOI:** 10.3390/polym15061576

**Published:** 2023-03-22

**Authors:** Fuping Wang, Guangyan Du, Chenggen Zhang, Qian-You Wang

**Affiliations:** 1 Department of Chemistry and Material Science, Langfang Normal University, Langfang 065000, China; 2 Henan Key Laboratory of Crystalline Molecular Functional Materials, Green Catalysis Center and College of Chemistry, Zhengzhou University, Zhengzhou 450001, China

**Keywords:** CL-20/TNT cocrystals, energetic materials, impact-sensitivity reduction, molecular dynamics

## Abstract

High-energy low-sensitivity explosives are research objectives in the field of energetic materials, and the formation of cocrystals is an important method to improve the safety of explosives. However, the sensitivity reduction mechanism of cocrystal explosives is still unclear. In this study, CL-20/TNT, CL-20 and TNT crystals were taken as research objects. On the basis of the ReaxFF-lg reactive force field, the propagation process of the wave front in the crystals at different impact velocities was simulated. The molecular dynamics data were used to analyze the molecular structure changes and initial chemical reactions, and to explore the sensitivity reduction mechanism of the CL-20/TNT cocrystal. The results showed that the chemical reaction of the CL-20/TNT cocrystal, compared with the CL-20 single crystal, is different under different impact velocities. At an impact velocity of 2 km/s, polymerization and separation of the component molecules weakened the decomposition of CL-20. At an impact velocity of 3 km/s, the decay rates of CL-20 and TNT in the cocrystal decreased, and the intermediate products were enhanced, such as nitrogen oxides. At an impact velocity of 4 km/s, the cocrystal had little effect on the decay rates of the molecules and formation of CO_2_, but it enhanced formation of N_2_ and H_2_O. This may explain the reason for the impact-sensitivity reduction of the CL-20/TNT cocrystal.

## 1. Introduction

High energy and low sensitivity are the goal in the field of energetic materials [1,2]. Hexanitrohexaazaisowurtzitane (CL-20) is a high-energy-density explosive [3], and reducing its sensitivity has become a current research hotspot. In 2011, Bolton et al. [4] prepared and obtained CL-20/2,4,6-trinitrotoluene (TNT) cocrystal energetic materials, which successfully reduced the impact sensitivity of CL-20. Subsequently, a large number of CL-20 cocrystals were successfully synthesized [5]. Through experimental tests and characterization of the material samples, it has been confirmed that the sensitivity of most CL-20 cocrystal energetic materials is significantly reduced and the safety is improved compared with CL-20 [6,7,8,9].

These exciting experimental phenomena have inspired researchers to explore the underlying reasons. Yang et al. [10] proposed that the formation of intermolecular forces and hydrogen bonds promotes close packing between CL-20 and TNT molecules, resulting in a reduction in the volume and number of cavities and the possibility of the formation of “hot spots”. Hu et al. [11] believed that the sensitivity decrease in CL-20/TNT is related to the spherical structure, which reduces the possibility of hot-spot formation. The molecular dynamics results of Hang et al. [12] showed that the intermolecular interaction energy, the energy required to trigger N–N bond breaking and the bonding strength of CL-20/TNT are greater than those of pure CL-20. Guo et al. [13] suggested that the carbon-rich clusters formed by TNT decomposition play an important role in the sensitivity reduction of CL-20/TNT, and the molecular steric hindrance also affects the anisotropy of the sensitivity [14]. Ren et al. [15] found that during thermal decomposition of CL-20/TNT, the interaction between TNT and the intermediate products delays the chain reaction, and the decomposition kinetics of CL-20 change [16]. Zhang et al. [17] found that under a constant shock-wave velocity, the initial decomposition steps in CL-20/TNT are the same as those of the components. The heat released by CL-20 decomposition is transferred to TNT, thus reducing its own decomposition rate. Xue et al. [18] believe that the decrease in the sensitivity of CL-20/HMX is related to the transfer of the heat released by CL-20 decomposition and the enhancement of intermolecular interactions.

Although many studies have been performed from multiple perspectives, the main reason for the decrease in the sensitivity of CL-20 cocrystals compared with CL-20 needs to be further analyzed, owing to the complex molecular structure and violent response under external stimulation [19,20,21]. In this study, we simulated the propagation process of the wave front in CL-20/TNT, CL-20 and TNT crystals at different impact velocities by molecular dynamics based on the ReaxFF-lg reactive force field. The changes of the molecular structures and initial chemical reactions were analyzed, and the mechanism for the decrease in the sensitivity is then discussed. This is the first study that focuses on the real propagation process of the wave front to analyze the sensitivity reduction mechanism of CL-20/TNT. The aim is to provide a deep understanding of the impact initiation mechanism and sensitivity reduction mechanism of CL-20/TNT cocrystals.

## 2. Calculation Methods and Simulation Details

**Figure 1:**
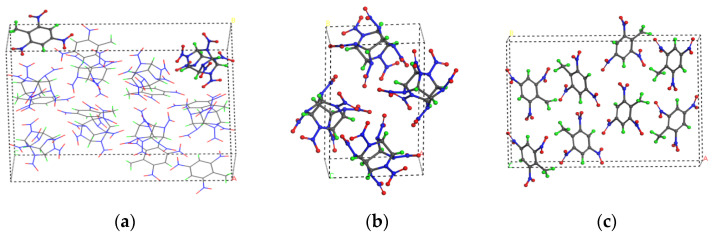
Unit-cell structures of the (**a**) CL-20/TNT, (**b**) CL-20 and (**c**) TNT crystals. (C, H, O and N atoms are shown in grey, green, red and blue, respectively.).

From the results of X-ray single-crystal diffraction experiments, the unit-cell structures of CL-20/TNT [4], CL-20 [22] and TNT [23] crystals were obtained (Figure 1). Based on the ReaxFF-lg reactive force field, the stable minimum-energy structures of the crystals were obtained by geometric optimization. Subsequently, canonical ensemble (NVT) and isobaric-isothermal (NPT) simulations were performed with a time step of 0.1 fs. The relaxation times were 10 and 15 ps at room temperature and zero pressure at room temperature, respectively.

**Figure 2:**
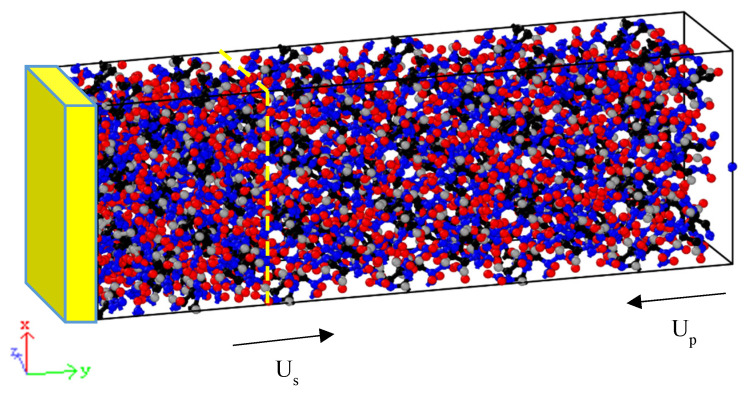
Schematic diagram of the shock-loading crystal calculation model (the left yellow region represents the reflection wall, Up represents the atomic directional velocity and Us represents the wave front propagation velocity). C, H, O, and N atoms are colored black, gray, red, and blue, respectively.

The supercell with a stable structure under normal temperature and pressure was expanded to construct a supercell with large spatial scale. One side of the supercell boundary was set as a fixed reflection wall, giving all of the atoms additional directional velocity to impact the reflection wall. Owing to the reflection wall, a wave array with a velocity gradient can be formed in the crystal and propagate along the opposite direction of the impact. A schematic diagram of the shock-loading crystal calculation model is shown in Figure 2.

The supercell was divided into multiple lamellas along the *y* direction, and the lamellas were formed into a unit-cell length with thickness in the *b* direction. At the initial time, all of the atoms were given a directional velocity of 2, 3 or 4 km/s along the −*y* direction to impact the reflector and generate shock waves. The microcanonical ensemble (NVE) was used for the whole shock process. The time step was 0.1 fs, and the connection between the atoms was output every 10 fs. The simulation was stopped before the wave front reached the right boundary. Processing and analysis of the molecular dynamics data were performed with a self-written program.

## 3. Results and Discussion

### 3.1. Verify the Reliability of Calculation Results

To verify the suitability of the force field, the lattice parameters and the density of the three relaxed crystals at 298 K and 0 Pa were compared with the initial structure from the experiments (as shown in Table 1). It is not difficult to find that the simulation results in this paper are very close to the experimental values. In addition, many researchers have successfully studied the chemical reaction of CL-20 [18,19], TNT [13] and CL-20/TNT [14] crystals using ReaxFF-lg reactive force field.

We also built three systems: the unit cell of TNT (eight TNT molecules), the unit cell of CL-20/TNT (eight TNT molecules and eight CL-20 molecules) and two unit cells of CL-20 (eight CL-20 molecules). The energy of each system is minimized under the ReaxFF-lg force field to obtain the energy value, as shown in Table 2. Sum the energy of CL-20 and TNT and compare with the CL-20/TNT. It is found that the energy of CL-20/TNT cocrystal molecules is lower than the sum of CL-20 and TNT. The explanation for this is that the formation of the cocrystal is not the superposition of the energies of two molecules, but it is more stable than the simple mixing of two molecules. This may be directly related to the sensitivity reduction in the CL-20/TNT cocrystal compared with CL-20.

**Figure 3:**
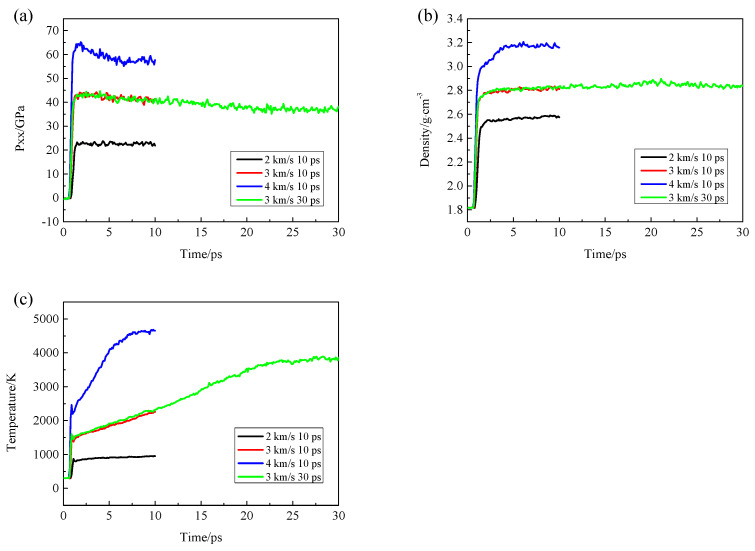
Change of pressure (**a**), density (**b**) and temperature (**c**) of CL-20/TNT cocrystal with time under different impact velocities.

A larger model was established and simulated for 30 ps at the impact velocity of 3 km/s in order to prove the repeatability of our results. The thermodynamic properties, such as temperature, pressure and density, were compared at different impact velocities (as shown in Figure 3). The red and green lines are all obtained at the impact velocity of 3 km/s. The red line is for 10 ps, and the green line is for 30 ps. The two curves fit well. Change of pressure, density and temperature of CL-20 and TNT with time under different impact velocities are in the Supplementary Materials (see Figure S1).

### 3.2. Changes in the Species

**Figure 4:**
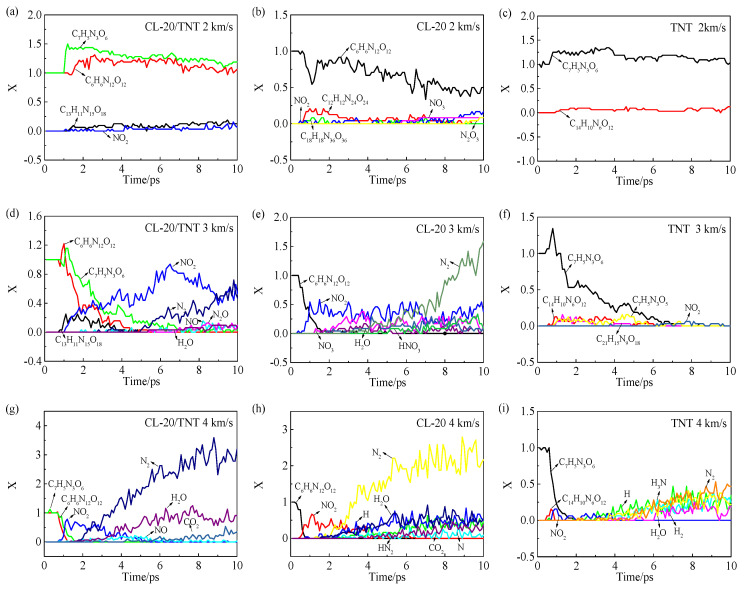
Time-dependent curves of the X values of the different substances in the CL-20/TNT, CL-20 and TNT crystals at impact velocities of (**a**–**c**) 2 km/s, (**d**–**f**) 3 km/s and (**g**–**i**) 4 km/s.

The unit cell of CL-20/TNT contains eight CL-20/TNT cocrystal molecules, the unit cell of CL-20 contains four CL-20 molecules (C_6_H_6_N_12_O_12_) and the unit cell of TNT contains eight TNT molecules (C_7_H_5_N_3_O_6_). To facilitate comparison, *X* is defined as the ratio of the number of molecules of the different species in a cell to the number of explosive molecules in a cell. The variation curves of the *X* values of the different substances in the CL-20/TNT, CL-20 and TNT crystals with time under different impact velocities are shown in Figure 4. The columns correspond to the CL-20/TNT, CL-20 and TNT crystals, and the rows correspond to impact velocities of 2, 3 and 4 km/s. 

From the perspective of the decomposition degree, at an impact velocity of 2 km/s, the species and number of small molecules generated by cocrystal decomposition were less than those of the CL-20 crystal, and the TNT crystal did not undergo a decomposition reaction. This indicates that TNT is less susceptible to chemical reaction than the CL-20/TNT cocrystal and the CL-20 crystal under impact, and thus its sensitivity is low. This conclusion is consistent with experimental results, which observed that the sensitivities of TNT and CL-20/TNT are lower than CL-20 [4]. It was worth noting that intermolecular polymerization occurs in all three crystals, but only the polymerization between CL-20 and TNT molecules occurred in the CL-20/TNT cocrystal. It can be speculated that the addition of TNT molecules in space and the strong interactions or hydrogen bonds between CL-20 and TNT molecules hindered the polymerization between two CL-20 molecules. Thus, the sensitivity of the CL-20/TNT cocrystal was reduced. 

In terms of the species, the main products in the CL-20/TNT cocrystal at different impact velocities were NO_2_, N_2_, NO, H_2_O, N_2_O and CO_2_. For the CL-20 crystal, the main products were NO_2_, NO_3_, H_2_O, HNO_3_, N_2_, HN_2_ and CO_2_. For the TNT crystal, C_7_H_5_N_3_O_5_ (as seen in Figure 4f), NO_2_, H_3_N, N_2_, H_2_O and H_2_ (as seen in Figure 4i) were mainly generated. The experimental results showed that NO_2_, N_2_O, NO and CO_2_ were the thermal decomposition products of CL-20 [24,25,26], and the detonation products were N_2_, H_2_O, CO_2_, CO and H_2_ [27]. Ornellas et al. [28] found that the detonation products of TNT were CO, H_2_O, N_2_, CO_2_, H_2_ and NH_3_. It can be concluded that our results are generally similar to the species measured in the experiment, but there are also differences. The reason for the differences may be related to the reaction conditions and the time scales.

The decay time is defined as the time taken for the explosive molecules to completely disappear. From the decay rates of the reactants, at an impact velocity of 3 km/s, the decay times of the molecules in the CL-20 and TNT crystals were 3 and 6.2 ps, respectively. The CL-20 and TNT molecules in the cocrystal completely disappeared after 4.6 and 9.1 ps, respectively. This indicates that after CL-20 and TNT formed the cocrystal, their decay rates were prolonged. 

At an impact velocity of 4 km/s, the decay times of the molecules in the CL-20 and TNT crystals were 0.6 and 1.2 ps, respectively. It takes 0.6 and 1.1 ps for CL-20 and TNT molecules in the cocrystal to completely disappear, respectively, which indicates that the molecular decay rates of the cocrystal and single crystals were almost the same at 4 km/s impact velocity. In short, the decay rate of CL-20 was greater than that of TNT under these two high-velocity impacts. 

From the quantity, at the impact velocity of 3 km/s, the amount of NO_2_ generated by the CL-20/TNT cocrystal was much larger than those of the other two crystals. Among the small-molecule products, the number of N_2_ molecules was the largest for the CL-20 crystal, and the number of C_7_H_5_N_3_O_5_ molecules was the largest for the TNT crystal. Therefore, cocrystal decomposition is helpful for the formation of intermediate products, such as NO_2_, NO and N_2_O, while the CL-20 crystal easily produces the N_2_ final product. At an impact velocity of 4 km/s, the numbers of N_2_ and H_2_O molecules in the CL-20/TNT cocrystal were larger than those in the CL-20 crystal, while the number of CO_2_ molecules was not significantly different from that in the CL-20 crystal. 

In summary, at the impact velocity of 3 km/s, CL-20 and TNT molecules in cocrystal decreased slowly, and the intermediate products increased. This may be related to the interaction between explosive molecules and intermediate products as well as heat transfer. At the impact velocity of 4 km/s, the desensitization mechanism of cocrystal was not obvious.

### 3.3. Analysis of the Bonds

To further analyze the formation and fracture of the chemical bonds, the bond breaking ratio *Y* is defined as follows:

$$Y=\frac{Number\;of\;bond\;generation-Number\;of\;bond\;breakage}{Maximum\;number\;of\;generated\;or\;broken}$$

A *Y* value of greater than zero indicates net generation of that type of chemical bond, and a *Y* value of less than zero indicates net fracture of that type of chemical bond. The numbers of the most generated types of chemical bonds in the CL-20/TNT, CL-20 and TNT crystals are given in Table 3.

**Figure 5:**
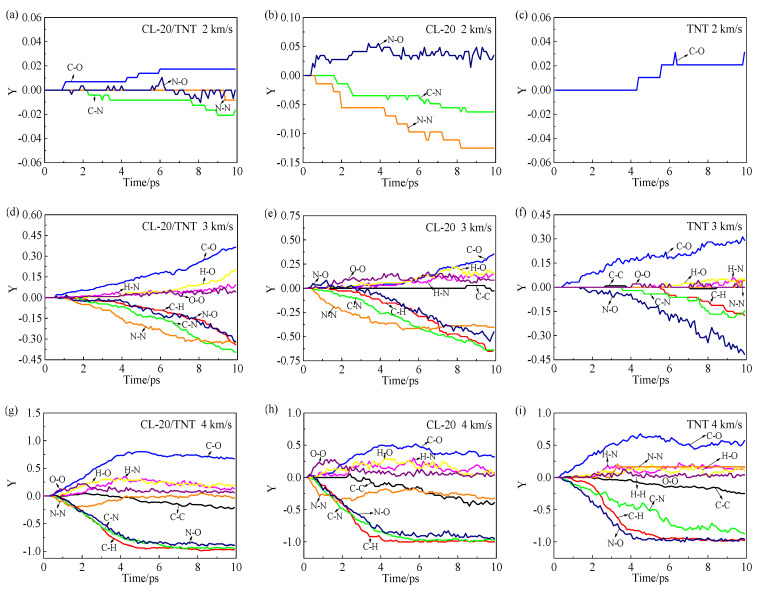
Formation and fracture numbers of the different types of chemical bonds in the CL-20/TNT, CL-20 and TNT crystals with time at impact velocities of (**a**–**c**) 2 km/s, (**d**–**f**) 3 km/s and (**g**–**i**) 4 km/s.

The changes of the *Y* values of the different types of chemical bonds for the CL-20/TNT, CL-20 and TNT crystals with time under different impact velocities are shown in Figure 5. For the CL-20/TNT cocrystal and for high impact velocity (3 and 4 km/s) in the CL-20 crystal, the N–O bond first formed and was then broken, and then other types of chemical bonds were generated or broken. The net generated chemical bonds were mainly C–O, O–O, H–N, H–O, H–H and N–N, and the net fractured chemical bonds were mainly C–N, N–N, C–H and C–C.

From the *Y* values, the formation and fracture ratios of the chemical bonds in the three crystals increased for higher impact velocity. Under the different impact velocities, various types of chemical bonds were most likely to be generated or broken in the CL-20 crystal, followed by the CL-20/TNT cocrystal, and finally the TNT crystal. This reflects the lower impact sensitivity of the cocrystal compared to the CL-20 crystal from a microscale perspective.

To investigate the initial chemical reaction, the results in Figure 5 were analyzed in depth, and the initial changes of the chemical bond types in the CL-20/TNT, CL-20 and TNT crystals at different impact velocities are summarized in Table 4. For the different impact velocities, the chemical bond types of each crystal are arranged in chronological order from top to bottom. At the same impact velocity, fewer bond changes shown in italics occurred in the CL-20/TNT cocrystal, indicating that the changes of chemical bond types of the cocrystal are consistent with those of CL-20. At the different impact velocities, the main initial reactions of the cocrystal were C–O formation, C–N fracture, N–O formation and N–N fracture. The main initial reactions of CL-20 were N–O generation, N–N fracture and C–N fracture. The main initial reactions of TNT were C–O formation, N–O fracture and C–N fracture. Although the types of chemical bonds were roughly the same, it does not mean that the reaction paths were the same.

### 3.4. Elementary Reactions

To determine the reaction path, we counted the elementary reactions that occurred earliest and with high frequency (see Tables S1–S3 in the Supplementary Materials). The earliest and high-frequency chemical reactions in the CL-20/TNT, CL-20 and TNT crystals at different impact velocities are given in Table 5. The earliest reactions in the three crystals at different impact velocities included polymerization of two or more molecules, which is due to the decrease in the intermolecular distance caused by compression of the impact wave front. The earliest decomposition reaction in the three crystals was direct decomposition of CL-20 or TNT to NO_2_. It is worth mentioning that the NO_2_ molecule in the cocrystal came from TNT at an impact velocity of 2 km/s and CL-20 at impact velocities of 3 and 4 km/s.

In the high-frequency reaction, at an impact velocity of 2 km/s, the three crystals were still dominated by polymerization. Besides polymerization, NO_2_ is generated from the decomposition of CL-20. At an impact velocity of 3 km/s, in the CL-20/TNT cocrystal, decomposition of CL-20 to NO_2_ and the reaction between NO_2_ molecules mainly occurred, as well as formation of N_2_O and N_2_. In the CL-20 crystal, generation of N_2_O_2_ from O_2_ and N_2_ mainly occurred, as well as the reaction between nitrogen oxides and hydroxide. Intermolecular polymerization and separation mainly occurred in the TNT crystal. At an impact velocity of 4 km/s, for the cocrystal, combination of N_2_ and H, and O and H_2_ mainly occurred, and the small molecules involved in the reaction were mainly N_2_, H_2_, H, O, HO and HN. The reactions between N_2_ and H or HO; O and H or H_2_; and H and HO mainly occurred in the CL-20 crystal. For the TNT crystal, the reactions were N with N, HN, H_3_ or H_4_, and the reaction between O and H_2_.

Through analysis of the impact velocity, it was found that at a low impact velocity of 2 km/s, the earliest and high-frequency reactions of the three crystals were polymerization and decomposition. At a medium impact velocity of 3 km/s, the reactions of CL-20/TNT and CL-20 were mainly related to nitrogen oxides. At a high impact velocity of 4 km/s, reactions between N and H or hydroxide mainly occurred in the three crystals.

### 3.5. Initial Reaction Steps

**Figure 6:**
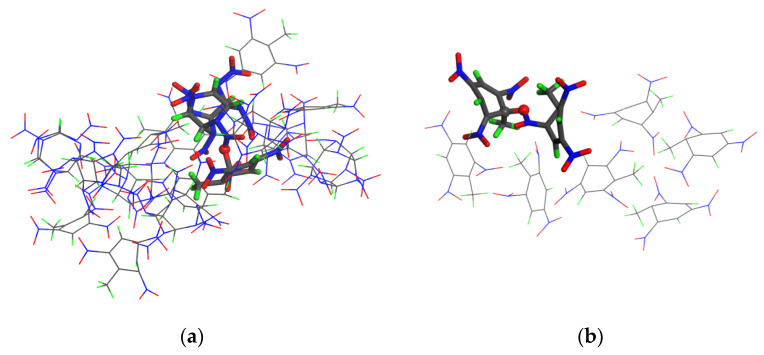
Molecular structure snapshots of the (**a**) CL-20/TNT (3 km/s 0.7 ps) and (**b**) TNT crystals (3 km/s 0.9 ps). C, H, O, and N atoms are colored gray, green, red, and blue, respectively.

Elementary reaction analysis showed that the three crystals first polymerized and then began to decompose owing to compression of the shock wave. In a previous study, we found that the CL-20 crystal under impact first forms a copolymer through combination of N and O, and it then breaks to form small NO_2_ molecules [29]. To determine which atoms were bonded in the polymerization reactions of the CL-20/TNT and TNT crystals, the molecular structure snapshots were searched and compared. The molecular structures of polymerization were found (Figure 6). Polymerization between the molecules in the CL-20/TNT and TNT crystals occurred through the C–O bond. This conclusion is consistent with the analysis results of the breaking bonds in Section 3.3.

**Figure 7:**
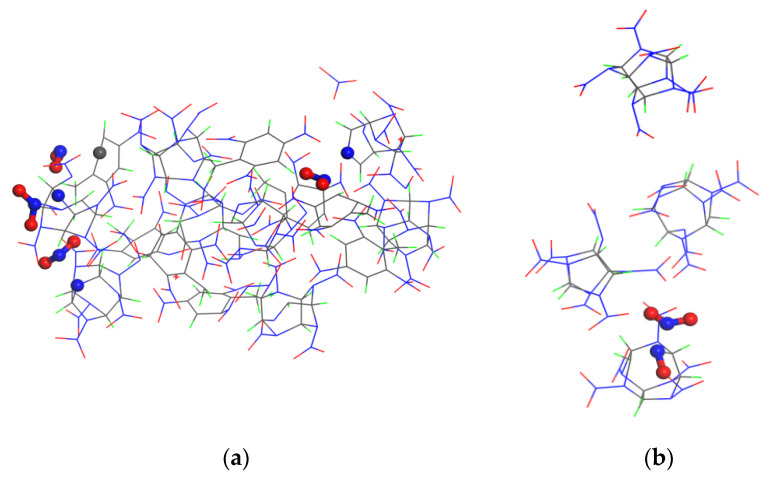
Molecular structure snapshots of the (**a**) CL-20/TNT cocrystal (3 km/s 2.0 ps) and (**b**) CL-20 crystal (2 km/s 0.8 ps). C, H, O and N atoms are shown in grey, green, red and blue, respectively.

According to the above species analysis, NO_2_ was the main initial decomposition product of the CL-20/TNT cocrystal and CL-20 crystal. Snapshots of the beginning of the appearance of NO_2_ molecules in the CL-20/TNT cocrystal and CL-20 crystal are shown in Figure 7. From Figure 7a, formation of NO_2_ in the cocrystal was mainly caused by direct cleavage of N–N bonds by CL-20 or TNT molecules, and most of the NO_2_ molecules came from the decomposition of CL-20 molecules. In Figure 7b, the N atom originally connected to NO_2_ was bonded to the O atom, and the N-NO_2_ bond was broken at the same time. Therefore, it can be concluded that NO_2_ in the CL-20 crystal was formed by attack of the O atom on the N atom, and this resulted in the fracture of the N–NO_2_ bond.

## 4. Conclusions

The propagation process of the wave front in CL-20/TNT, CL-20 and TNT crystals at different impact velocities was simulated by molecular dynamics, and the molecular structure changes and initial chemical reactions were analyzed. Under impact, the CL-20/TNT cocrystal and TNT crystal formed polymers by C–O bonds, and then the N–N bond broke to form NO_2_. NO_2_ in the cocrystal mainly came from decomposition of CL-20. The chemical reaction of the CL-20/TNT cocrystal, compared with the CL-20 single crystal, is different under different impact velocities. When the CL-20/TNT cocrystal was impacted at low velocity (2 km/s), polymerization and separation of the component molecules easily occurred, which weakened the decomposition of the CL-20 molecules. At a medium impact velocity (3 km/s), the decay rates of CL-20 and TNT in the cocrystal decreased, prolonging the primary reaction process and promoting formation of the intermediate products (such as nitrogen oxides). At a high impact velocity (4 km/s), the cocrystal had little effect on the decay rates of the molecules and formation of CO_2_, but it enhanced the formation of N_2_ and H_2_O. This may explain the reason for the impact-sensitivity reduction of the CL-20/TNT cocrystal.

## Figures and Tables

**Table 1:** Comparison of lattice parameters and density of three relaxed crystals.

Crystals	Methods	a/Å	b/Å	c/Å	Density/g·cm^−3^
CL-20/TNT	Experiments	9.674	19.369	24.690	1.910
	ReaxFF-lg	9.836	19.693	25.103	1.818
CL-20	Experiments	8.863	12.593	13.395	2.035
	ReaxFF-lg	9.038	12.842	13.660	1.919
TNT	Experiments	20.041	15.013	6.084	1.648
	ReaxFF-lg	19.689	14.750	5.977	1.738

**Table 2:** Energy comparison of CL-20, TNT and CL-20/TNT crystals.

.	CL-20	TNT	Sum	CL-20/TNT	∆
Energy (Kcal/mol)	−29,224.383	−20,096.508	−49,320.891	−49,363.63	42.739

**Table 3:** Numbers of the most generated chemical bonds in CL-20/TNT, CL-20 and TNT.

Crystals	Bonds Styles									
	C-C	C-H	C-N	C-O	H-H	H-N	H-O	N-N	N-O	O-O
CL-20/TNT	10	11	15	18	5.5	11	11	7.5	18	9
CL-20	3	6	12	12	3	6	6	6	12	6
TNT	7	5	3	6	2.5	5	5	1.5	6	3

**Table 4:** Initial changes of the chemical bond types in the CL-20/TNT, CL-20 and TNT crystals at different impact velocities *.

Impact Velocities/km·s^−1^	CL-20/TNT	CL-20	TNT
2	*C-O generation*	**N-O generation**	*C-O generation*
	**C-N broken**	**N-N broken**	−
	**N-O generation**	**C-N broken**	−
	**N-N broken**	−	−
3	C-O generation	**N-O generation**	C-O generation
	**N-O generation**	**N-N broken**	*N-O broken*
	C-N broken	C-N broken	C-N broken
	H-N generation	**O-O generation**	*C-H broken*
	**N-N broken**	C-O generation	*H-O generation*
	*C-H broken*	−	−
	**O-O generation**	−	−
4	**N-O generation**, C-N broken	**N-O generation**	−
	C-O generation	**O-O generation, N-N broken**, C-H broken	C-O generation
	**O-O generation, N-N broken**	**H-N generation**, C-N broken, C-O generation	*N-O broken*
	C-H broken	H-O generation	*C-C broken*, C-N broken
	*C-C broken*	*C-C broken*	C-H broken

* The changes of the bond types in the CL-20 crystal are shown in bold, the changes in the bond types in the TNT crystal are shown in italics and the changes that occurred in all three crystals are shown in red.

**Table 5:** Earliest and high-frequency chemical reactions in the CL-20/TNT, CL-20 and TNT crystals at different impact velocities.

Impact Velocities /km·s^−1^	Crystal Species	First Occur	Highest Frequency
2	CL-20/TNT	Polymerization of component molecules	Polymerization and separation between component molecules
		Decomposition of TNT to NO_2_	
	CL-20	Bimolecular polymerization	Multi-molecular polymerization
			CL-20 decomposition to NO_2_
	TNT	Bimolecular or multi-molecular polymerization	Intermolecular polymerization and separation
3	CL-20/TNT	Polymerization of component molecules	NO_2_+NO_2_→N_2_O_4_
		CL-20 decomposition to NO_2_	CL-20 decomposition to NO_2_
		—	Generation of N_2_O, N_2_
	CL-20	Bimolecular polymerization	Bimolecular polymerization
		CL-20 decomposition to NO_2_	O_2_+N_2_→N_2_O_2_
			NO, HO
	TNT	Bimolecular or multi-molecular polymerization	Intermolecular polymerization and separation
4	CL-20/TNT	Polymerization of component molecules	N_2_ and H
		CL-20 decomposition to NO_2_	O and H_2_
	CL-20	Bimolecular polymerization	N_2_ and H or HO
		CL-20 decomposition to NO_2_	O and H or H_2_
			H and HO
	TNT	Bimolecular or multi-molecular polymerization	N and N, HN, H_3_ or H_4_
			O and H_2_

## Data Availability

All data generated or analyzed during this study are included in this published article.

## Supplementary Materials

The following supporting information can be downloaded at https://www.mdpi.com/article/10.3390/polym15061576/s1, Figure S1: Change of pressure, density and temperature of CL-20 and TNT with time under different impact velocities; Table S1: Elementary reactions of CL-20/TNT decomposition at different impact velocities. Table S2: Elementary reactions of CL-20 decomposition at different impact velocities. Table S3: Elementary reactions of TNT decomposition at different impact velocities.
