# Supplementary material for: Mechanism of the Impact-Sensitivity Reduction of Energetic CL-20/TNT Cocrystals: A Nonequilibrium Molecular Dynamics Study
Source: Polymers (Basel). 2023 Mar 22;15(6):1576. doi: 10.3390/polym15061576 (PMC10057516; doi:10.3390/polym15061576)
Supplement: Supplementary file 1 [file polymers-15-01576-s001.zip › polymers-2230110-supplementary.pdf]

Mechanism of the impact-sensitivity reduction of energetic CL-20/TNT cocrystals: a nonequilibrium molecular dynamics study

Fuping Wang[1,*], Guangyan Du[1], Chenggen Zhang[1] and Qian-You Wang[2]

[1] Department of Chemistry and Material Science, Langfang Normal University, Langfang, 065000, China
[2] Henan Key Laboratory of Crystalline Molecular Functional Materials, Green Catalysis Center and College of Chemistry, Zhengzhou University, Zhengzhou 450001, China
* Correspondence: wangfuping@lfnu.edu.cn

Supplementary Materials:

Figure S1: Change of pressure, density and temperature of CL-20 and TNT with time under different impact velocities; Table S1: Elementary reactions of CL-20/TNT decomposition at different impact velocities. Table S2: Elementary reactions of CL-20 decomposition at different impact velocities. Table S3: Elementary reactions of TNT decomposition at different impact velocities.

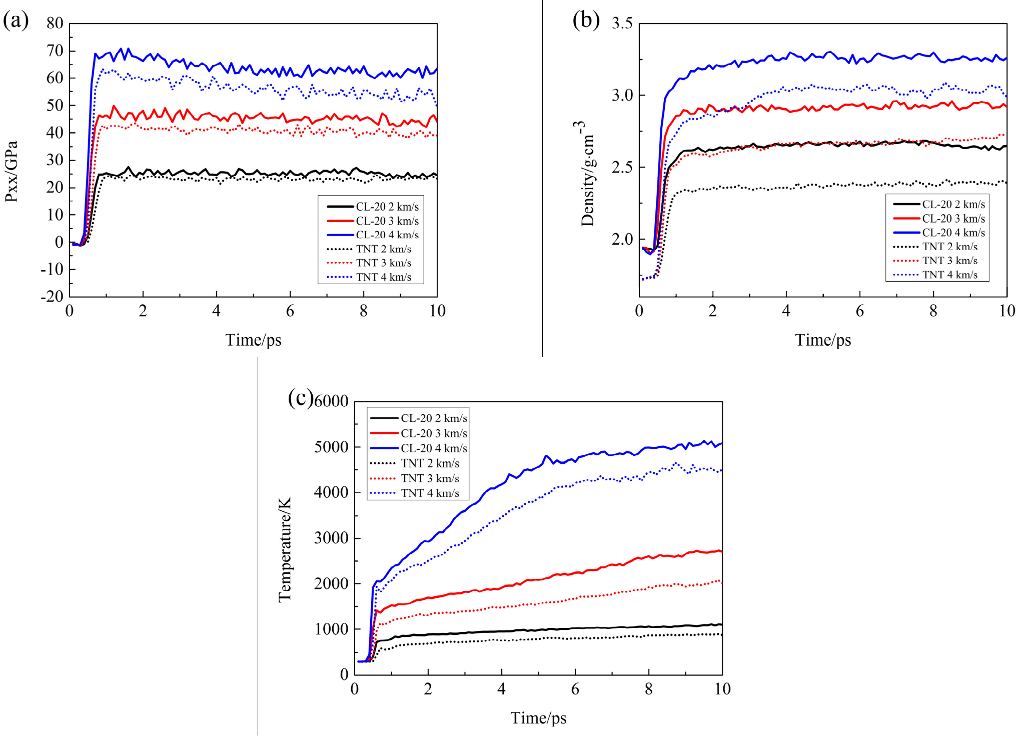

Figure S1. Change of pressure, density and temperature of CL-20 and TNT with time under different impact velocities.

Table S1. Elementary reactions of CL-20/TNT cocrystal decomposition at different impact velocities.

Impact velocities /km/s	Frequencies	Reaction time/ps	Elementary reactions	Illustration
	4	1.0-6.0	$\mathbf{C_6H_6N_{12}O_{12}+C_7H_5N_3O_6 \rightarrow C_{13}H_{11}N_{15}O_{18}}$	
	2	1.9-7.1	$\mathbf{C_{13}H_{11}N_{15}O_{18}+C_6H_6N_{12}O_{12} \rightarrow C_{19}H_{17}N_{27}O_{30}}$	
2	1	2.0-2.0	$\mathbf{C_{19}H_{17}N_{27}O_{30} \rightarrow C_{13}H_{11}N_{15}O_{18}+C_6H_6N_{12}O_{12}}$	First occur
	2	2.7-5.9	$\mathbf{C_7H_5N_3O_6 \rightarrow C_7H_5N_3O_6\text{-}X(C_6H_6N_{12}O_{12})}$	
	1	3.0-3.0	$C_7H_5N_3O_6\text{-}X(C_6H_6N_{12}O_{12}) \rightarrow \mathbf{NO_2}+C_7H_5N_2O_4\text{-}X(C_6H_6N_{12}O_{12})$	

	1	3.1-3.1	$NO_2+C_7H_5N_2O_4\text{-}X(C_6H_6N_{12}O_{12})\rightarrow C_7H_5N_3O_6\text{-}X(C_6H_6N_{12}O_{12})$	
	4	1.0-6.0	**$C_6H_6N_{12}O_{12}+C_7H_5N_3O_6\rightarrow C_{13}H_{11}N_{15}O_{18}$**	Highest frequency
	3	3.8-5.9	**$C_6H_6N_{12}O_{12}\rightarrow C_6H_6N_{12}O_{12}\text{-}X(C_6H_6N_{12}O_{12})$**	
	3	3.9-6.0	**$C_6H_6N_{12}O_{12}\text{-}X(C_6H_6N_{12}O_{12})\rightarrow C_6H_6N_{12}O_{12}$**	
	3	7.9-9.8	$C_7H_5N_5O_8\rightarrow$ **N_2**$+C_7H_5N_3O_8$	
	3	7.8-8.7	$N_2+C_7H_5N_3O_8\rightarrow C_7H_5N_5O_8$	
3	2	0.5-0.8	**$C_6H_6N_{12}O_{12}\rightarrow C_6H_6N_{12}O_{12}\text{-}X(C_6H_6N_{12}O_{12})$**	First occur
	1	0.6-0.6	**$C_6H_6N_{12}O_{12}\text{-}X(C_6H_6N_{12}O_{12})\rightarrow C_6H_6N_{12}O_{12}$**	
	1	0.6-0.6	$C_6H_6N_{12}O_{12}+C_6H_6N_{12}O_{12}+C_6H_6N_{12}O_{12}+C_6H_6N_{12}O_{12}+C_7H_5N_3O_6+C_7H_5N_3O_6\rightarrow$ **$C_{38}H_{34}N_{54}O_{60}$**	
	1	0.7-0.7	**$C_7H_5N_3O_6\rightarrow C_7H_5N_3O_6\text{-}X(C_6H_6N_{12}O_{12})$**	
	1	0.7-0.7	$C_{38}H_{34}N_{54}O_{60}\rightarrow$ **NO_2**$+C_{19}H_{17}N_{26}O_{28}+C_{19}H_{17}N_{27}O_{30}$	
	4	0.7-1.4	$C_6H_6N_{12}O_{12}\rightarrow C_6H_6N_{12}O_{12}\text{-}X(C_7H_5N_3O_6)$	
	2	0.8-0.8	$C_6H_6N_{12}O_{12}\text{-}X(C_7H_5N_3O_6)\rightarrow C_6H_6N_{12}O_{12}$	
	4	4.1-8.5	**$NO_2\text{-}X(NO_2)\rightarrow NO_2$**	Highest frequency
	4	4.0-8.2	**$NO_2\rightarrow NO_2\text{-}X(NO_2)$**	
	3	2.8-7.3	$NO_2+NO_2\rightarrow$ **N_2O_4**	
	3	1.7-3.0	$C_6H_6N_{12}O_{12}\rightarrow$ **NO_2**$+C_6H_6N_{11}O_{10}$	
	2	7.5-7.0	$O\text{-}X(N_2O_3)+N_2\rightarrow$ **N_2O**$\text{-}X(N_2O_3)$	
	2	6.4-7.2	$NO_2\text{-}X(HO)\rightarrow NO_2$	
	2	5.9-6.1	$C_7H_5N_6O_{11}\rightarrow$ **N_2**$+C_7H_5N_4O_{11}$	
	2	3.9-6.0	$N_2O_4\rightarrow NO_2+NO_2$	
	2	3.6-4.2	$C_{13}H_{11}N_{14}O_{17}\rightarrow NO_2+C_{13}H_{11}N_{13}O_{15}$	
	2	2.3-2.9	$C_7H_5N_4O_8\rightarrow NO_2+C_7H_5N_3O_6$	
	2	2.2-2.7	$NO_2+C_7H_5N_3O_6\rightarrow C_7H_5N_4O_8$	
	2	2.0-2.3	$NO_2+C_6H_6N_{12}O_{12}\rightarrow C_6H_6N_{13}O_{14}$	
	2	1.8-2.6	$C_{13}H_{11}N_{14}O_{17}+C_6H_6N_{11}O_9\rightarrow C_{19}H_{17}N_{25}O_{26}$	
	2	1.6-2.4	$C_7H_5N_3O_6\rightarrow C_7H_5N_3O_6\text{-}X(NO_2)$	
	2	1.3-1.5	$C_{13}H_{11}N_{15}O_{18}\rightarrow NO_2+C_{13}H_{11}N_{14}O_{16}$	
4	2	0.5-0.5	**$C_6H_6N_{12}O_{12}+C_7H_5N_3O_6\rightarrow C_{13}H_{11}N_{15}O_{18}$**	First occur
	2	0.5-0.5	**$C_6H_6N_{12}O_{12}\rightarrow NO_2+C_6H_6N_{11}O_{10}$**	
	2	4.3-9.9	$N_2\rightarrow N_2\text{-}X(H)$	
	2	5.2-9.6	$O\text{-}X(H_2)\rightarrow O\text{-}X(H)$	
	2	5.2-9.9	$HO\text{-}X(H)\rightarrow HO$	
	2	6.1-9.4	$N_2\text{-}X(H)\rightarrow N_2$	
	28	4.5-9.9	**$N_2\text{-}X(H)\rightarrow N_2$**	Highest frequency
	21	4.3-9.9	**$N_2\rightarrow N_2\text{-}X(H)$**	
	11	5.2-9.6	**$O\text{-}X(H_2)\rightarrow O\text{-}X(H)$**	
	10	5.1-8.7	**$O\text{-}X(H_2)\rightarrow O\text{-}X(H_2)$**	
	10	2.8-9.3	**$O\text{-}X(H)\rightarrow O\text{-}X(H_2)$**	
	9	5.7-9.7	$N\text{-}X(HN)\rightarrow N\text{-}X(N)$	
	9	4.4-8.2	$HO\rightarrow HO\text{-}X(H)$	
	8	7.7-9.8	$H\text{-}X(N_2)\rightarrow H\text{-}X(HO)$	
	8	5.2-9.9	$HO\text{-}X(H)\rightarrow HO$	
	8	3.8-9.7	$N_2\rightarrow N_2\text{-}X(HO)$	
	7	5.7-9	$H\text{-}X(HO)\rightarrow H\text{-}X(HO)$	
	6	7.8-9.9	$H\text{-}X(HO)\rightarrow H\text{-}X(N_2)$	
	6	5.8-7.4	$N_2\text{-}X(H_2)\rightarrow N_2\text{-}X(H)$	

Table S2. Elementary reactions of CL-20 decomposition at different impact velocities.

Impact velocities	Frequencies	Reaction time/ps	Elementary reactions	Illustration

/km/s				
2	9	0.3-4.1	$C_6H_6N_{12}O_{12} \rightarrow C_6H_6N_{12}O_{12}\text{-X}(C_6H_6N_{12}O_{12})$	First occur
	2	0.5-0.7	$C_6H_6N_{12}O_{12} + C_6H_6N_{12}O_{12} \rightarrow C_{12}H_{12}N_{24}O_{24}$	
	1	0.5-0.5	$C_6H_6N_{12}O_{12}+C_6H_6N_{12}O_{12}\text{-X}(C_6H_6N_{12}O_{12}) \rightarrow C_{12}H_{12}N_{24}O_{24}\text{-X}(C_6H_6N_{12}O_{12})$	
	1	0.6-0.6	$C_6H_6N_{12}O_{12}\text{-X}(C_6H_6N_{12}O_{12}) \rightarrow C_6H_6N_{12}O_{12}\text{-X}(C_{12}H_{12}N_{24}O_{24})$	
	1	0.6-0.6	$C_{12}H_{12}N_{24}O_{24} \rightarrow C_6H_6N_{12}O_{12}+C_6H_6N_{12}O_{12}$	
	5	0.7-8.2	$C_6H_6N_{12}O_{12} \rightarrow$ **NO_2**$+C_6H_6N_{11}O_{10}$	
	13	2.7-9.5	**$C_6H_6N_{11}O_{10} \rightarrow C_6H_6N_{11}O_{10}\text{-X}(C_6H_6N_{12}O_{12})$**	Highest frequency
	12	3.4-9.1	**$C_6H_6N_{11}O_{10}\text{-X}(C_6H_6N_{12}O_{12}) \rightarrow C_6H_6N_{11}O_{10}$**	
	9	0.3-4.1	**$C_6H_6N_{12}O_{12} \rightarrow C_6H_6N_{12}O_{12}\text{-X}(C_6H_6N_{12}O_{12})$**	
	6	0.8-4.2	**$C_6H_6N_{12}O_{12}\text{-X}(C_6H_6N_{12}O_{12}) \rightarrow C_6H_6N_{12}O_{12}$**	
	5	0.7-8.2	$C_6H_6N_{12}O_{12} \rightarrow$ **NO_2**$+C_6H_6N_{11}O_{10}$	
	3	5.1-6.4	$C_6H_6N_{12}O_{12} \rightarrow C_6H_6N_{12}O_{12}\text{-X}($**$C_{24}H_{24}N_{47}O_{45}$**$)$	
	3	5.0-6.3	$C_6H_6N_{12}O_{12}\text{-X}(C_{24}H_{24}N_{47}O_{45}) \rightarrow C_6H_6N_{12}O_{12}$	
	3	2.2-2.9	$C_6H_6N_{12}O_{12} \rightarrow C_6H_6N_{12}O_{12}\text{-X}(C_{42}H_{42}N_{83}O_{82})$	
3	6	0.3-0.5	$C_6H_6N_{12}O_{12} \rightarrow C_6H_6N_{12}O_{12}\text{-X}(C_6H_6N_{12}O_{12})$	First occur
	4	0.4-0.7	$C_6H_6N_{12}O_{12}\text{-X}(C_6H_6N_{12}O_{12}) \rightarrow C_6H_6N_{12}O_{12}$	
	1	0.4-0.4	$C_6H_6N_{12}O_{12}+ C_6H_6N_{12}O_{12} \rightarrow C_{12}H_{12}N_{24}O_{24}$	
	1	0.5-0.5	$C_6H_6N_{12}O_{12}+C_6H_6N_{12}O_{12}\text{-X}(C_6H_6N_{12}O_{12}) \rightarrow C_{12}H_{12}N_{24}O_{24}\text{-X}(C_6H_6N_{12}O_{12})$	
	1	0.5-0.5	$C_6H_6N_{12}O_{12}\ C_6H_6N_{12}O_{12} \rightarrow C_{12}H_{12}N_{24}O_{24}\text{-X}(C_6H_6N_{12}O_{12})$	
	1	0.5-0.5	$C_{12}H_{12}N_{24}O_{24} \rightarrow$ **NO_2** $+C_{12}H_{12}N_{23}O_{22}\text{-X}(C_6H_6N_{12}O_{12})$	
	6	0.3-0.5	**$C_6H_6N_{12}O_{12} \rightarrow C_6H_6N_{12}O_{12}\text{-X}(C_6H_6N_{12}O_{12})$**	Highest frequency
	4	7.5-9.7	**$O_2+N_2 \rightarrow N_2O_2$**	
	4	0.4-0.7	$C_6H_6N_{12}O_{12}\text{-X}(C_6H_6N_{12}O_{12}) \rightarrow C_6H_6N_{12}O_{12}$	
	3	7.6-8.1	$N_2O_2 \rightarrow O_2+N_2$	
	2	8.0-9.3	$NO\text{-X}($**HO_2**$) \rightarrow NO\text{-X}($**$O_2$**$)$	
	2	6.9-9.9	$N_2 \rightarrow N_2\text{-X}($**$HO$**$)$	
	2	6.3-8.2	$NO \rightarrow NO\text{-X}($**$H$**$)$	
	2	6.3-7.2	$NO_4\text{-X}(HN_2O_3) \rightarrow NO_4\text{-X}(NO)$	
	2	6.3-6.7	$NO_2\text{-X}($**NO_3**$) \rightarrow NO_2$	
	2	5.9-8.7	$NO\text{-X}(O) \rightarrow NO\text{-X}(HNO_4)$	
	2	5.0-9.7	$NO_2 \rightarrow NO_2\text{-X}(H)$	
	2	4.2-4.6	$NO_2 +NO_3\text{-X}(H) \rightarrow N_2O_5\text{-X}(H)$	
	2	3.7-5.0	$O\text{-X}($**N_2O_4**$) \rightarrow O\text{-X}(NO_2)$	
	2	2.5-2.8	$NO_2 \rightarrow NO_2\text{-X}($**$N_2O_5$**$)$	
	2	1.8-3.4	$NO_2 \rightarrow NO_2\text{-X}(NO_3)$	
4	1	0.2-0.2	$C_6H_6N_{12}O_{12} \rightarrow C_6H_6N_{12}O_{12}\text{-X}(C_{18}H_{18}N_{32}O_{28})$	First occur
	1	0.2-0.2	$C_6H_6N_{12}O_{12}+C_6H_6N_{12}O_{12}+C_{12}H_{12}N_{24}O_{24}\text{-X}($**$C_{198}H_{198}N_{380}O_{353}$**$)$	
	1	0.3-0.3	$C_6H_6N_{12}O_{12} \rightarrow C_6H_6N_{12}O_{12}\text{-X}($**$O$**$)$	
	1	0.3-0.3	$C_{12}H_{12}N_{24}O_{24}\text{-X}(C_{198}H_{198}N_{380}O_{353}) +C_6H_6N_{12}O_{12}+C_6H_6N_{12}O_{12} +C_6H_6N_{12}O_{12}+C_6H_6N_{12}O_{12}+C_6H_6N_{12}O_{12}+C_6H_6N_{12}O_{12}+C_6H_6N_{12}O_{12}\text{-X}(C_{18}H_{18}N_{32}O_{28}) \rightarrow$ **NO_2+NO_2**$+C_{12}H_{12}N_{23}O_{23}\text{-X}(C_6H_6N_{12}O_{12})+C_{12}H_{12}N_{24}O_{23}\text{-X}(C_{12}H_{12}N_{24}O_{22})+C_{30}H_{30}N_{59}O_{58}\text{-X}($**$C_{242}H_{240}N_{453}O_{441}$**$)$	
	1	0.4-0.4	$C_{12}H_{12}N_{23}O_{23}\text{-X}(C_6H_6N_{12}O_{12})+C_{12}H_{12}N_{24}O_{23}\text{-X}(C_{12}H_{12}N_{24}O_{22}) +C_{30}H_{30}N_{59}O_{58}\text{-X}(C_{242}H_{240}N_{453}O_{441})+ C_6H_6N_{12}O_{12} +C_6H_6N_{12}O_{12} +C_6H_6N_{12}O_{12}\text{-X}(O) \rightarrow O\text{-X}(C_6H_6N_{12}O_{11})$ +**NO_2**+ **NO_2**+ **NO_2** +**NO_2** $+C_6H_6N_{10}O_8 +C_{66}H_{66}N_{128}O_{123}\text{-X}(C_{347}H_{342}N_{645}O_{611})$	
	1	0.5-0.5	$NO_2+ C_6H_6N_{10}O_8 \rightarrow NO_3\text{-X}(H) +C_6H_6N_{10}O_7$	
	40	4.2-9.9	$N_2 \rightarrow N_2\text{-X}(H)$	Highest frequency
	26	4.1-9.8	**N_2**$\text{-X}($**H**$) \rightarrow N_2$	

22	3.0-9.4	$N_2\text{-}X(\mathbf{HO}) \rightarrow N_2$
16	2.8-9.9	$N_2 \rightarrow N_2\text{-}X(HO)$
15	3.4-9.0	$O\text{-}X(H) \rightarrow O\text{-}X(H_2)$
10	3.8-9.5	$O\text{-}X(\mathbf{H_2}) \rightarrow O\text{-}X(H)$
9	3.9-9.9	$O\text{-}X(H_2) \rightarrow O\text{-}X(H_2)$
8	3.9-8.6	$N_2\text{-}X(H) \rightarrow N_2\text{-}X(H_2)$
8	3.5-9.1	$H\text{-}X(HO) \rightarrow H\text{-}X(HO)$
8	1.7-9.5	$N_2 \rightarrow N_2\text{-}X(N_2)$
7	5.7-9.7	$N_2\text{-}X(H_2) \rightarrow N_2$
7	4.7-9.9	$N\text{-}X(\mathbf{HN}) \rightarrow N\text{-}X(N)$

Table S3. Elementary reactions of TNT decomposition at different impact velocities.

Impact velocities /km/s	Frequencies	Reaction time/ps	Elementary reactions	Illustration
2	4	2.2-6.6	$\mathbf{C_7H_5N_3O_6 \rightarrow C_7H_5N_3O_6\text{-}X(C_7H_5N_3O_6)}$	First occur
	3	4.4-6.3	$\mathbf{C_7H_5N_3O_6+C_7H_5N_3O_6 \rightarrow C_{14}H_{10}N_6O_{12}}$	
	2	4.5-4.8	$C_7H_5N_3O_6\text{-}X(C_7H_5N_3O_6) \rightarrow C_7H_5N_3O_6$	
	1	5.3-5.3	$C_7H_5N_3O_6 \rightarrow C_7H_5N_3O_6\text{-}X(\mathbf{C_{21}H_{15}N_9O_{18}})$	
	4	2.2-6.6	$\mathbf{C_7H_5N_3O_6 \rightarrow C_7H_5N_3O_6\text{-}X(C_7H_5N_3O_6)}$	Highest frequency
	3	4.4-6.3	$\mathbf{C_7H_5N_3O_6 + C_7H_5N_3O_6 \rightarrow C_{14}H_{10}N_6O_{12}}$	
	2	4.5-4.8	$\mathbf{C_7H_5N_3O_6\text{-}X(C_7H_5N_3O_6) \rightarrow C_7H_5N_3O_6}$	
3	8	0.4-1.2	$C_7H_5N_3O_6 \rightarrow C_7H_5N_3O_6\text{-}X(C_7H_5N_3O_6)$	First occur
	3	0.5-0.9	$C_7H_5N_3O_6\text{-}X(C_7H_5N_3O_6) \rightarrow C_7H_5N_3O_6$	
	1	0.5-0.5	$C_7H_5N_3O_6+C_7H_5N_3O_6 \rightarrow C_{14}H_{10}N_6O_{12}$	
	1	0.6-0.6	$C_7H_5N_3O_6 \rightarrow C_7H_5N_3O_6\text{-}X(C_{14}H_{10}N_6O_{12})$	
	1	0.6-0.6	$C_{14}H_{10}N_6O_{12}+C_7H_5N_3O_6 \rightarrow C_{21}H_{15}N_9O_{18}$	
	1	0.7-0.7	$C_{21}H_{15}N_9O_{18} \rightarrow C_{21}H_{15}N_9O_{18}\text{-}X(C_{14}H_{10}N_6O_{12})$	
	1	0.9-0.9	$C_{21}H_{15}N_9O_{18}\text{-}X(O)+C_7H_5N_3O_6 \rightarrow C_{28}H_{20}N_{12}O_{24}X(\mathbf{O})$	
	8	0.4-1.2	$C_7H_5N_3O_6 \rightarrow C_7H_5N_3O_6\text{-}X(C_7H_5N_3O_6)$	Highest frequency
	3	0.5-0.9	$C_7H_5N_3O_6\text{-}X(C_7H_5N_3O_6) \rightarrow C_7H_5N_3O_6$	
4	1	0.3-0.3	$C_7H_5N_3O_6 + C_7H_5N_3O_6 \rightarrow C_{14}H_{10}N_6O_{12}$	First occur
	3	0.3-0.4	$C_7H_5N_3O_6 \rightarrow C_7H_5N_3O_6\text{-}X(C_7H_5N_3O_6)$	
	1	0.4-0.4	$C_{14}H_{10}N_6O_{12} \rightarrow C_{14}H_{10}N_6O_{12}\text{-}X(\mathbf{C_{347}H_{238}N_{144}O_{293}})$	
	1	0.4-0.4	$C_7H_5N_3O_6+C_7H_5N_3O_6\text{-}X(C_7H_5N_3O_6) \rightarrow C_{14}H_{10}N_6O_{12}\text{-}X(C_7H_5N_3O_6)$	
	1	0.4-0.4	$C_7H_5N_3O_6+C_7H_5N_3O_6 \rightarrow C_{14}H_{10}N_6O_{12}\text{-}X(C_{14}H_{10}N_6O_{12})$	
	1	0.5-0.5	$C_{14}H_{10}N_6O_{12}\text{-}X(C_{347}H_{238}N_{144}O_{293}) \rightarrow C_{14}H_{10}N_6O_{12}\text{-}X(\mathbf{C_{385}H_{266}N_{158}O_{313}})$	
	5	6.7-9.2	$N\text{-}X(\mathbf{N}) \rightarrow N\text{-}X(HN)$	Highest frequency
	5	3.4-7.9	$O\text{-}X(\mathbf{H_2}) \rightarrow O\text{-}X(H)$	
	3	8.3-9.7	$O\text{-}X(H_2) \rightarrow O\text{-}X(H_2)$	
	3	8.0-9.7	$N\text{-}X(\mathbf{H_3}) \rightarrow N\text{-}X(H_2)$	
	3	5.9-8.8	$N_2 \rightarrow N_2\text{-}X(H)$	
	3	5.8-9.5	$N\text{-}X(\mathbf{HN}) \rightarrow N\text{-}X(H_2N)$	
	3	5.8-8.1	$N\text{-}X(\mathbf{H_4}) \rightarrow N\text{-}X(H_3)$	
